# The diagnosis and molecular epidemiology investigation of avian hepatitis E in Shandong province, China

**DOI:** 10.1186/s12917-021-03079-2

**Published:** 2022-01-25

**Authors:** Kuihao Liu, Yiran Zhao, Jun Zhao, Ningwei Geng, Fanliang Meng, Siqi Wang, Jing Li, Zhaobing Zhong, Liya Zhu, Sidang Liu, Ning Li

**Affiliations:** 1 College of Animal Science and Technology, Shandong Agricultural University, Sino-German Cooperative Research Centre for Zoonosis of Animal Origin Shandong Province, Shandong Provincial Key Laboratory of Animal Biotechnology and Disease Control and Prevention, Shandong Provincial Engineering Technology Research Center of Animal Disease Control and Prevention, 61 Daizong Road, Taian, 271000 Shandong Province China; 2 Taian Daiyue District Administrative Examination and Approval Service Bureau, Taian, 271018 Shandong Province China; 3 Animal Husbandry and Veterinary Service Centre of Linshu, Linyi, 276700 Shandong Province China

**Keywords:** Avian hepatitis E virus, Mixed infections, Potential novel genotype, Molecular epidemiology

## Abstract

**Background:**

Avian hepatitis E virus (HEV) is the pathogenic agent of big liver and spleen disease (BLS) and of hepatitis-splenomegaly syndrome (HSS) in chickens, which have caused economic losses to the poultry industry in China. In this study, 18 samples of BLS chickens were collected to reveal the molecular epidemiological characteristics of avian HEV in the province of Shandong, China.

**Results:**

Gross and microscopic lesions of clinical samples were observed; then, virology detection and genetic analysis of avian HEV were performed. The results showed that there was significant swelling and rupture in the liver and that the spleen was enlarged. Microscopic lesions demonstrated obvious hemorrhage in the liver, with infiltration of heterophilic granulocytes, lymphocytes, and macrophages, as well as the reduction of lymphocytes in the spleen. Eleven of the 18 samples were positive for avian HEV, with a positive rate of 61.11%. More importantly, all avian HEV-positive samples were mixed infections: among these, the mixed infections of avian HEV and chicken infectious anemia virus (CIAV) and avian HEV and fowl adenovirus (FAdV) were the most common. Furthermore, the genetic evolution analysis showed that all avian HEV strains obtained here did not belong to the reported 4 genotypes, thus constituting a potential novel genotype.

**Conclusions:**

These results of this study further enrich the epidemiological data on avian HEV in Shandong, prove the genetic diversity of avian HEV in China, and uncover the complex mixed infections of avian HEV clinical samples.

**Supplementary Information:**

The online version contains supplementary material available at 10.1186/s12917-021-03079-2.

## Background

Avian hepatitis E virus (HEV) can cause big liver and spleen disease (BLS) and hepatitis-splenomegaly syndrome (HSS) in chickens, which are characterized by hepatosplenomegaly. Avian HEV primarily infects laying hens and broiler breeders through fecal-oral transmission [1], resulting in decreased laying rates and increased mortality [2]. Since the first avian HEV strain was isolated and identified in China in 2010 [3], the diseases caused by avian HEV have become increasingly prevalent in chickens in recent years, causing substantial economic losses to the chicken industry in China.

The Hepatitis E virus belong to the genus *Orthohepevirus*, which contains four species, designated as *Orthohepevirus* A, *Orthohepevirus* B, *Orthohepevirus* C, and *Orthohepevirus* D. Avian HEV is a non-enveloped, single-stranded, positive sense RNA virus belonging to *Orthohepevirus* B [4, 5]. Its genome is approximately 6.6 kb and contains three open reading frames (ORFs) and 3′ and 5′ non-coding regions. Of these, the capsid protein encoded by ORF2 is highly conserved and has been extensively used in viral genotyping and genetic evolution analysis of avian HEV [6].

Recently, the outbreak of hepatosplenomegaly disease has increased in chickens; although avian HEV is considered to be the major causative agent, fowl adenovirus (FAdV), reticuloendotheliosis virus (REV), avian leukosis virus (ALV), Marek’s disease virus (MDV), and chicken infectious anemia virus (CIAV) can also cause hepatosplenomegaly and immunosuppression, which may facilitate the spread of avian HEV [6, 7]. Therefore, continual epidemiological investigation of avian HEV is necessary. In the present study, suspected cases of avian HEV infection were diagnosed and the molecular epidemiology of avian HEV in chickens was characterized in the province of Shandong between 2020 and 2021. These results can enhance the current understanding of the genetic diversity of avian HEV and provide new insights into prevention and control strategies of this disease.

## Results

### Gross and microscopic lesions

**Fig. 1:**
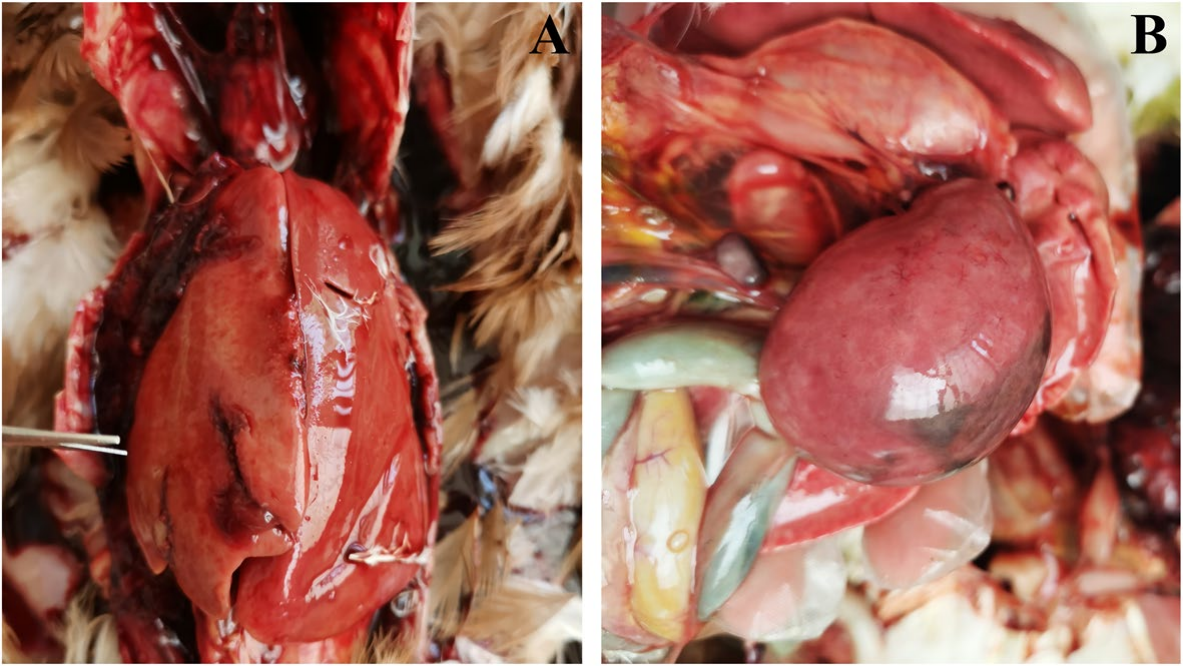
Gross lesions of HEV-infected chickens. **A** Hemorrhage and swelling of the liver, **B** Splenomegaly

**Fig. 2:**
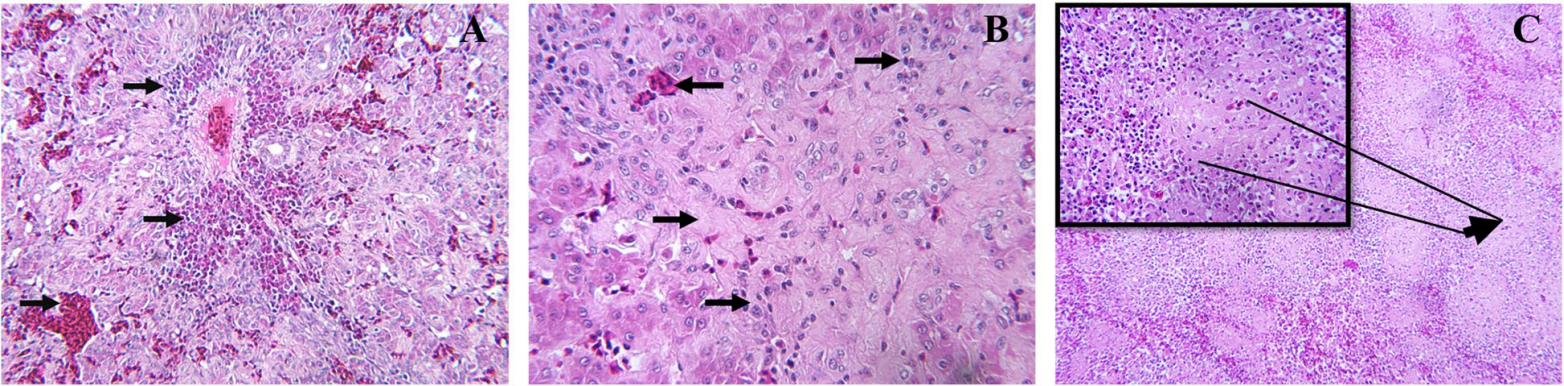
Histopathological lesions of avian HEV-infected chickens. **A** Extensive hemorrhagic foci in liver tissue, massive infiltration of portal areas with lymphocytes and heterophils. Amplification 200×; **B** Degeneration and necrosis of liver cells, massive amyloid deposition, and infiltration of red blood cells and macrophage. Amplification 400×; **C** The decreased lymphocyte and extensive amyloid deposition in the spleen. Amplification 40 ×

The major gross lesions of suspected cases of avian HEV were concentrated in the liver and spleen. In the collected samples, significant enlargement, rupture, and bleeding spots of the liver were found (Fig. 1A), and the spleen was enlarged with spots of bleeding and necrosis foci on the surface (Fig. 1B). Histopathological lesions showed hepatocellular necrosis and hemorrhagic foci in liver tissue, with massive heterophil and lymphocyte infiltration around portal areas (Fig. 2A), necrosis of liver cells and amyloid deposition with a small amount of red blood cells and macrophage infiltration (Fig. 2B), and reduced numbers of lymphocytes in the spleen and extensive amyloid deposition (Fig. 2C).

### Virus detection

PCR detection revealed that 11 of the 18 samples had avian HEV infection, with a positive rate of 61.11%. The 11 avian HEV-positive strains were located in the cities of Taian (2), Dezhou (2), Zibo (2), Heze (2), Linyi (1), and Jining (2), and the ages of diseased chickens were 18–25 weeks old. Interestingly, all positive samples were mixed infections, with 2 cases of avian HEV and CIAV co-infection, 1 case of avian HEV and FAdV co-infection, 5 cases of triple infection, and 3 cases of quadruple infection (Table 1). In addition, virus infections were also observed in another 5 samples; these were mixed infections of CIAV, FAdV, and MDV. No viruses were detected in the remaining 2 samples.

**Table 1:** The mixed infections of avian HEV and other viruses

Type of mixed infection	Number of mixed infection cases	Viruses
Co-infection	2	avian HEV, CIAV
	1	avian HEV, FAdV
Triple infection	4	avian HEV, CIAV, FAdV
	1	avian HEV, CIAV, MDV
Quadruple infection	3	avian HEV, CIAV, FAdV, MDV

### Seque nce homology analysis and phylogenetic tree construction

The partial ORF2 gene of the 11 avian HEV strains was amplified for sequencing and alignment. The results showed that the nucleotide homology across the 11 samples was 96.3–100%, and the homology of amino acids was 96.2–100%. The highest nucleotide homology was observed between the 11 avian HEV strains and others belonging to *Orthohepevirus* B (75.6–83.5%). However, the homologies with *Orthohepevirus* A, *Orthohepevirus* C, and *Orthohepevirus* D were low, ranging from 48.2 to 56.7%. Consequently, we further analyzed the nucleotide homology between the 11 avian HEV strains and the different genotype viruses belonging to *Orthohepevirus* B. We found in this study that these strains had low homology with the 4 reported genotypes, ranging from 75.6 to 83.5%, among which the homology with genotype 1 was the highest (78.9–83.5%) (Table 2). The nucleotide and amino acid homologies between strains isolated in this study and potential novel genotypes that have been reported recently (accession No. MG976720, MG692744, MH094852 and MN562265) were 74.6–86.0% and 83.8–100%, respectively.

**Table 2:** The nucleotide and amino acid homologies between the 11 avian HEV strains and reference strains with different genotypes

Strains	Different genotypes of avian HEV^a^ (% identity)				
	Genotype 1	Genotype 2	Genotype 3	Genotype 4	
1–11	78.9–83.5	75.6–79.8	81.4–83.1	78.5–79.8	nucleotide
	95.0–97.5	93.8–97.5	96.2–98.8	95.0–97.5	amino acid

^a^ The reference viruses of the different genotypes are shown in supplementary Table 1

**Fig. 3:**
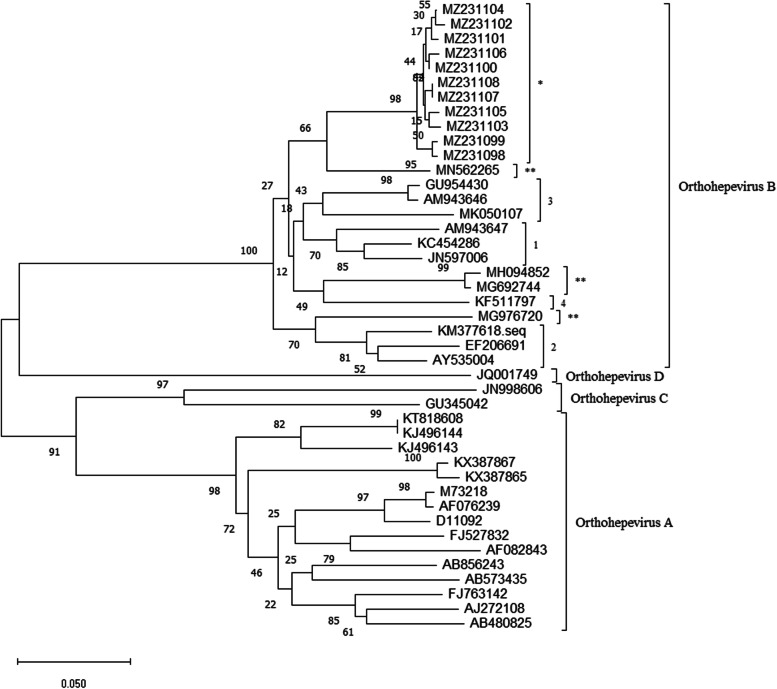
The tree was constructed by the neighbor joining method with 1000 bootstrap replicates, using MEGA 6.0. The phylogenetic tree was constructed based on the partial ORF2 gene of avian HEV (242 bp) and 30 reference strains found in NCBI. The detailed information of the reference strains are shown in supplementary Table 1. Strains in this study are marked with asterisk (*), while potential novel genotypes reported in other articles are marked with double asterisks (**)

The 11 avian HEV strains obtained in this study have been submitted to NCBI (accession No. MZ231098 to MZ231108), and the phylogenetic tree was constructed with other known avian HEV virus strains of different genotypes. The results demonstrate that the 11 strains did not share a branch with any known genotypes, and they constituted a single branch (Fig. 3).

## Discussion

Avian HEV was first reported in 1991 in western Canada and subsequently caused outbreaks in the United States, Australia, and the United Kingdom [8, 9]. It has been reported that severe avian HEV infections have occurred in several provinces in China since 2016 [3, 10, 11]. In recent years, outbreaks of BLS and HSS in chicken flocks have gradually increased in the province Shandong [10, 12]. In order to investigate the causes of BLS and HSS along with the epidemic characteristics of avian HEV in Shandong, 18 cases of hepatomegaly and splenomegaly were collected for histopathology examination and pathogen detection, the latter including avian HEV, MDV, FAdV, ALV, CIAV, and REV. Histopathological examination revealed hemorrhage and necrosis in the liver and that the heterophagic granulocytes and lymphocytes were infiltrated in the portal areas of the liver; this was similar to the results of lymphocytic phlebitis and periphlebitis of the liver in the artificial challenge chickens infected with avian HEV [1, 13]. Moreover, a reduction of lymphocytes and amyloidosis were observed in the spleen. However, due to the present lack of an efficient cell culture system for avian HEV isolation, the bile samples of chickens infected with avian HEV were used for animal challenge experiments, instead of purified virus [1, 13]. Therefore, the isolation of avian HEV strains and related animal experiments for the purpose of obtaining accurate results will be the focus of further research. Notably, there was a large amount of heterophagic granulocyte infiltration in the liver in this study; this type of cell is related to bacterial infection, indicating that avian HEV infections may cause secondary infections in clinical samples. The role of the mixed infections of multiple viruses in BLS and HSS cannot be ruled out.

Mixed infections of avian HEV and ALV [6] and of avian HEV and MDV have been reported since 2016 [14], and the mixed infection rate of avian HEV and several immunosuppressive viruses was up to 58% in chickens in China; co-occurring viruses were CIAV, ALV, and REV [7], indicating that the mixed infections of avian HEV and other viruses is common in chicken flocks. In the current study, PCR detection showed that all 11 avian HEV-positive samples were mixed infections, of which the co-occurring infection rates of avian HEV and CIAV (90.90%) and of avian HEV and FAdV (72.72%) were highest. CIAV is a critical chicken immunosuppressive virus: according to Li et al.’s epidemiological survey of chicken vertical transmission or immunosuppressive virus co-infection, the detection rate of CIAV is the highest (26.5%) [15]. Meanwhile, FAdV has been prevalent in poultry in recent years and has caused huge economic losses to the poultry industry in China [16, 17]. It has been reported that chickens infected with FAdV were more likely to be infected with immunosuppressive diseases [18], and a previous study also reported that the co-infection rate of avian HEV and FAdV was about 10% [7]. The problem of vaccines contaminated with CIAV and FAdV may facilitate the serious level of mixed-infection rates [19]. However, the role that CIAV and FAdV have played in the pathogenesis of avian HEV, and whether they can promote the onset of avian HEV, are questions worthy of further study.

A total of 18 samples were collected for this study, 11 of which tested positive for avian HEV and mixed infections with other viruses. It is worth noting that in two of the samples with clinical manifestations of HSS and BLS, we did not detect avian HEV or other viruses. There is still controversy regarding whether diseases characterized by HSS or BLS in chickens are completely caused by avian HEV [6, 20]. On the whole, this study confirmed the pervasiveness and severity of avian HEV and other viruses in chicken flocks, and continued epidemiological surveillance is required.

Different genotypes of avian HEV have been reported worldwide [10, 21–23]. Zhao et al. reported the complete genome sequence of the first avian HEV in 2010 [3]; since then, avian HEV strains of different genotypes have been reported widely in China, including in the provinces of Shandong, Jiangxi, and Guangdong [10, 13, 24]. In terms of the molecular characteristics of the 11 avian HEV strains, the nucleotide and amino acid homologies of all avian HEV strains were highest with reference strains belonging to *Orthohepevirus* B. Genetic evolution analysis demonstrated that all strains were located in a single branch and were different from the known 4 genotype strains, indicating a potential novel genotype. The avian HEV strains collected from different areas in Shandong had high nucleotide homology with each other, indicating that this potential novel genotype of avian HEV may be widespread in Shandong. It has been reported that avian HEV can be spread through vertical transmission [25], but further investigations are required as to whether such transmission can facilitate the spread of the potential novel genotype in different regions.

An unavoidable limitation of this study is the small number of samples. Therefore, we will expand the areas and the number of samples in our further research in order to enrich the epidemiological data.

## Conclusions

In conclusion, this study found a potential novel genotype of avian HEV in Shandong, enriched the molecular epidemiological data of avian HEV, and found significant serious mixed infections of avian HEV and other viruses in clinical samples.

## Methods

### Samples

In this study, 18 clinical cases (10 dead laying hens and 8 dead broiler breeders) showing severe hepatosplenomegaly, rupture, and bleeding were collected. These samples came from different farms located in the cities of Taian, Dezhou, Zibo, Liaocheng, Heze, Linyi, Jinan, and Jining in Shandong province. The ages of affected chickens ranged from 17 to 25 weeks, and there was a significant decrease in the laying, hatching, and survival rates of chicks. The livers and spleens were collected and divided into two parts. One part of the collected tissues (about 1 g each) was minced and diluted 1:10 in phosphate-buffered saline (PBS, pH 7.2), then subjected to freeze-thaw cycles three times; the supernatant was collected after 12,000-rpm centrifugation for 5 min and stored at − 80 °C for virus detection. The other part of the tissues was fixed with 10% formalin solution, routinely processed, and stained using the HE method for histopathological examination [26].

### Nucleic acid extraction and virus detection

Each sample’s liver and spleen were mixed and ground to extract nucleic acid. The nucleic acid (RNA/DNA) was extracted from the treated samples using the Simply P Virus DNA/RNA Extraction Kit (Bioer, Hangzhou, China), according to the procedures described in the manufacturer’s instructions. The obtained virus nucleic acids were divided into two parts: one part was reverse-transcribed into cDNA by ReverTra Ace qPCR RT Kit (TOYOBO, Shanghai, China) for the detection of RNA viruses—including avian HEV, ALV, and REV—and another part was used for the detection of DNA viruses (MDV, FAdV, and CIAV). Virus detection was conducted by the PCR method. All primers refer to previous research [16, 20] (Table 3). In line with the instructions of 2 × Accurate Taq Master Mix (Dye Plus) (Accurate Biotechnology, Hunan, China), the total volume of the reaction system was 20 μL, and the thermal cycling conditions were as follows: 94 °C for 30 s, 35 cycles of 98 °C for 10 s, 54–60 °C for 30 s, and 72 °C for 1 min, with a final incubation at 72 °C for 2 min.

**Table 3:** The primers used in the study

Primers	Sequence (5′-3′)	Product size (bp)	Annealing temperature (°C)
CIAV-F	CAGAATTCCCACCTCAAGCGACTTCGAC	580	54
CIAV-R	ATGTCGACGGGGCTGAAGGAT		
REV-F	CATACTGAGCCAATGGTT	300	54
REV-R	AATGTTGTAGCGAAGTACT		
MDV-F	TCATCAGGGTCTCCCGTCACCT	1005	55
MDV-R	AGAGATGTCTCAGGAGCCAGAG		
FAdV-F	AATTTCGACCCCATGACGCGCCAGG	508	56
FAdV-R	TGGCGAAAGGCGTACGGAAGTAAGC		
ALV-J-F	GGATGAGGTGACTAAGA	512	56
ALV-J-R	CGAACCAAAGGTAACACACG		
Avian HEV-F1	TCGCCT(C)GGTAAT(C)ACA(T)AATGC	278	60
Avian HEV-R1	GCGTTC(G)CCG(C)ACAGGT(C)CGGCC		
Avian HEV-F2	ACA(T)AATGCT(C)AGGGTCACCCG	242	56
Avian HEV-R2	ATGTACTGA(G)CCA(G)CTG(C)GCCGC		

### Phylogenetic analysis of avian HEV strains

The PCR-positive products were cloned to the pMD18-T vector (Takara, Dalian, China) and were sent to Sangon Biotech (Shanghai, China) for sequencing. Sequences of avian HEV strains generated in this study were submitted to the GenBank under accession numbers MZ231098–MZ231108. The phylogenetic and molecular evolutions of these avian HEV strains were analyzed using MEGA version 6 [27] by the neighbor joining method with 1000 bootstrap replicates, and the comparison of sequence identities was performed using MegAlign software (DNAStar, Madison, United States).

## Supplementary Information

**Additional file 1 Supplementary Table 1.** The information of reference strains in the study.

## Data Availability

The viral sequences obtained in this study are deposited in GenBank under the accession numbers: MZ231098 to MZ231108. The data involving in the manuscript can be obtained from the corresponding author upon reasonable request.

## Abbreviations

PCR: Polymerase chain reaction
PBS: Phosphate-buffered saline
Avian HEV: Avian hepatitis E virus
BLS: Big liver and spleen disease
HSS: Hepatitis-splenomegaly syndrome
FAdV: Fowl adenovirus
REV: Reticuloendotheliosis virus
ALV: Avian leukosis virus
MDV: Marek’s disease virus
CIAV: Chicken infectious anemia virus
HE: Hematoxylin-eosin staining
