# Supplementary material for: The diagnosis and molecular epidemiology investigation of avian hepatitis E in Shandong province, China
Source: BMC Vet Res. 2022 Jan 25;18:56. doi: 10.1186/s12917-021-03079-2 (PMC8788081; doi:10.1186/s12917-021-03079-2)
Supplement: Supplementary file 1 — Additional file 1 Supplementary Table 1. The information of reference strains in the study. [file 12917_2021_3079_MOESM1_ESM.docx]

**Additional file 1 The information of reference strains in the study.**

**Supplementary table 1. The information of reference strains in the study.**

| Accession number | Strain | Country of origin | Collection_date |
| --- | --- | --- | --- |
| AM943647 | 06-561 | Australia | 1986 |
| KC454286 | JY-F2 | South Korea | 2011 |
| JN597006 | HH-F9 | South Korea | 2011 |
| MK050107 | CaHEV-GDSZ01 | China | 2018 |
| GU954430 | CaHEV | China | 2009 |
| AM943646 | 05-5492 | Hungary:Europe | 2005 |
| KF511797 | KF511797 | Taiwan | 2012 |
| KM377618 | GI-B | South Korea | 2014 |
| EF206691 | Avirulent | USA | 2007 |
| AY535004 | Prototype | USA | 2018 |
| JQ001749 | BatHEV/BS7/GE/2009 | Germany | 2009 |
| JN998606 | FRHEV4 | Netherlands | 2010 |
| GU345042 | R63 | Germany | 2009 |
| KT818608 | Patient 17 | Singapore | 2011 |
| KJ496144 | 180C | United Arab Emirates | 2013 |
| KJ496143 | 178C | United Arab Emirates | 2013 |
| M73218 | M73218 | Burma | 2000 |
| AF076239 | Hyderabad | India: Hyderabad | 2001 |
| D11092 | D11092 | China | 2008 |
| FJ527832 | SAAS-JDY5 | China: Shanghai | 2009 |
| AF082843 | Prototype swine | USA | 1998 |
| AJ272108 | T1 | China | 2000 |
| AB480825 | HE-JF5/15F_p6 | Japan | 2009 |
| FJ763142 | KNIH-hHEV4 | South Korea | 2010 |
| AB856243 | wbJNN_13 | Japan:Nagano | 2013 |
| AB573435 | JBOAR135-Shiz09 | Japan:Shizuoka, Tenryu | 2009 |
| KX387867 | 62XJ | China: Xinjiang | 2016 |
| KX387865 | 12XJ | China: Xinjiang | 2020 |
| MN562265 | CHN-GS-aHEV | China | 2020 |
| MH094852 | PT16B | Pakistan | 2018 |
| MG692744 | PT12B | Pakistan | 2018 |
